# Predictive value of DNA repair gene expression for response to neoadjuvant chemotherapy in breast cancer

**DOI:** 10.1590/1414-431X2021e11857

**Published:** 2022-03-11

**Authors:** M.C. Kneubil, K.O.B. Goulart, J. Brollo, G.P. Coelho, J. Mandelli, B.C. Orlandin, L.L. Corso, M. Roesch-Ely, J.A.P. Henriques

**Affiliations:** 1 Instituto de Biotecnologia, Universidade de Caxias do Sul, Caxias do Sul, RS, Brasil; 2 Laboratório de Patologia Anatômica e Biologia Molecular, Grupo Diagnose, Caxias do Sul, RS, Brasil; 3 Hospital Geral, Universidade de Caxias do Sul, Caxias do Sul, RS, Brasil; 4 Área do Conhecimento de Ciências Exatas e Engenharias, Universidade de Caxias do Sul, Caxias do Sul, RS, Brasil; 5 Programas de Pós-Graduação em Biotecnologia e em Ciências Médicas, Universidade do Vale do Taquari - UNIVATES, Lajeado, RS, Brasil; 6 Instituto Hercílio Randon, Caxias do Sul, RS, Brasil

**Keywords:** Breast cancer, Neoadjuvant chemotherapy, Expression of DNA repair genes

## Abstract

Genome-wide analysis using microarrays has revolutionized breast cancer (BC) research. A substantial body of evidence supports the clinical utility of the 21-gene assay (Oncotype DX) and 70-gene assay (MammaPrint) to predict BC recurrence and the magnitude of benefit from chemotherapy. However, there is currently no genetic tool able to predict chemosensitivity and chemoresistance to neoadjuvant chemotherapy (NACT) during BC treatment. In this study, we explored the predictive value of DNA repair gene expression in the neoadjuvant setting. We selected 98 patients with BC treated with NACT. We assessed DNA repair expression in 98 formalin-fixed, paraffin-embedded core biopsy fragments used at diagnosis and in 32 formalin-fixed, paraffin-embedded post-NACT residual tumors using quantitative reverse transcription-polymerase chain reaction. The following genes were selected: *BRCA1*, *PALB2*, *RAD51C*, *BRCA2*, *ATM*, *FANCA*, *MSH2*, *XPA*, *ERCC1*, *PARP1*, and *SNM1*. Of 98 patients, 33 (33.7%) achieved pathologic complete response (pCR). The DNA expression of 2 genes assessed in pre-NACT biopsies (*PALB2* and *ERCC1*) was lower in pCR than in non-pCR patients (P=0.005 and P=0.009, respectively). There was no correlation between molecular subtype and expression of DNA repair genes. The genes *BRCA2* (P=0.009), *ATM* (P=0.004), *FANCA* (P=0.001), and *PARP1* (P=0.011) showed a lower expression in post-NACT residual tumor samples (n=32) than in pre-NACT biopsy samples (n=98). The expression of 2 genes (*PALB2* and *ERCC1*) was lower in pCR patients. These alterations in DNA repair could be considered suitable targets for cancer therapy.

## Introduction

Breast cancer (BC) is a heterogeneous group of neoplasms in terms of their molecular alterations (1). Specific biological processes, distinct genetic pathways, and different molecular subtypes are associated with different prognosis and sensitivity to treatment (2). Defective DNA repair pathways allow cancer cells to accumulate genomic alterations that contribute to their aggressive phenotype (3). These alterations induce genome instability and promote carcinogenesis steps, cancer progression, and chemoresistance (4,5).

The neoadjuvant setting provides a rich environment for the investigation of therapies and biomarkers (6). Chemotherapy-induced DNA damage is processed by several key pathways that work together to eliminate DNA lesions and maintain genome stability and integrity (4). There is strong emerging evidence that overexpression of DNA repair factors can contribute to resistance to cancer treatment (7).

Genome-wide analysis using microarrays has revolutionized the field of BC research, classifying breast cancer by gene expression profiling (8). In patients with BC, a substantial body of evidence supports the clinical utility of gene expression profiling. For example, the 21-gene assay (Oncotype DX^®^,USA) and 70-gene assay (MammaPrint^®^, The Netherlands) predict BC recurrence and the magnitude of benefit from chemotherapy (9,10). However, there is currently no gene-expression profiling able to predict chemosensitivity and chemoresistance to neoadjuvant chemotherapy (NACT) during BC treatment.

Therefore, the development of specific predictive biomarkers for chemoresistance and chemosensitivity is desirable. In the present study, we explored the predictive value of DNA repair gene expression for response to NACT in BC by evaluating the mRNA expression of 11 selected genes that have a key role in DNA repair mechanisms. In addition, *BRCA1, BRCA2, RAD51C, ATM*, and *PALB2* are tightly correlated with breast cancer and have been associated with breast cancer predisposition, clinicopathological features, and prognosis (11). On the other hand, *ERRC1, FANCA, MSH2, XPA,* and *SNM1A* have been well documented as markers of resistance to chemotherapy in solid tumors (7,12-15) Currently, *PARP1* is a therapeutic target in the treatment for patients with *BRCA1-* or *BRCA2*-mutated BC (16).

## Material and Methods

We retrospectively reviewed the medical records of patients with BC who received NACT from January 2012 to June 2020 at a private clinic and at a General Hospital.

We analyzed data on the patients' medical history, clinicopathological features, type of surgery, and NACT modality. We excluded patients who had distant metastases at diagnosis.

### Pathologic and subtype stratification

We assessed estrogen receptor (ER)/progesterone receptor (PgR) status, human epidermal growth factor receptor 2 (HER2) protein, and Ki-67 antigen with the following primary antibodies: monoclonal antibody (MAb) to ER (Dako, USA, clone EP1, prediluted), MAb to PgR (Dako, clone PgR, prediluted), MIB-1 MAb to Ki-67 antigen (Dako, clone MIB-1, prediluted), and polyclonal antiserum (Biogen, USA, clone SP3, 1/1100 dilution) to HER2 protein. Intense and complete membrane staining in >10% of the tumor cells qualified for HER2 immunohistochemical (IHC) expression (3+). For this analysis, HER2 scores of 0 and 1+ were considered negative. HER2 IHC 3+ and FISH-amplified tumors were considered positive. All IHC 2+ tumors and tumors for which IHC was not assessable were also tested for gene amplification by FISH. The value of Ki-67 labeling index was divided into low (<14%) and high (≥14%). According to the St. Gallen BC subtype approximations (2), we stratified BC into 5 tumor subtypes: 1) luminal A - ER- and/or PgR-positive, HER2-negative, and low Ki-67 (<14%); 2) luminal B/HER2-negative - ER- and/or PgR-positive, HER2-negative, and high Ki-67 (≥14%); 3) luminal B/HER2-positive - ER- and/or PgR-positive, any Ki-67, and HER2-positive; 4) non-luminal/HER2-positive - ER-negative, PgR-negative, and HER2-positive; and 5) triple negative - ER-negative, PgR-negative, and HER2-negative.

### Reverse transcription and gene expression

We selected the following genes for analysis: *BRCA1*, *PALB2*, *RAD51C*, *BRCA2*, *ATM*, *FANCA*, *MSH2*, *XPA*, *ERCC1*, *PARP1*, and *SNM1*. The analyses were developed as described by Cronin et al. (17) and Paik et al. (18) for the evaluation of gene expression in formalin-fixed, paraffin-embedded tumor tissue.

After performing hematoxylin and eosin (H&E) staining of a 3-µm slice from the original paraffin blocks by mirroring, a pathologist selected the area of exclusive tumor mass according to the morphological criteria of anatomopathological diagnosis of invasive breast carcinoma in the paraffin block, ensuring the purity of the tumor sample to the detriment of possible contamination of the paraffin sample used for extraction of genetic material. With a surgical blade, we dissected the areas of interest containing representative invasive carcinoma, excluding areas of *in situ* carcinoma, necrosis, and normal breast tissue. We removed the paraffin by xylene extraction and extracted RNA using the RecoverAll™ Total Nucleic Acid Isolation kit (Invitrogen-ThermoFisher™, USA), according to the manufacturer's instructions. Immediately after RNA extraction, we performed reverse transcription to obtain cDNA using the High-Capacity cDNA Reverse Transcription kit (Applied Biosystems-Thermo Fisher, USA). This material was frozen at −20°C until used in the quantitative reverse transcription-polymerase chain reaction (qRT-PCR) assay.

Before the amplification reaction, we quantified the cDNA with a Qubit^®^ 2.0 fluorometer using the Qubit^®^ dsDNA HS assay (Invitrogen-ThermoFisher™), according to the manufacturer's instructions. We then performed qRT-PCR with the 7500 Fast Real-Time PCR System using TaqMan^®^ Gene Expression and pre-designed TaqMan^®^ probes (all from Applied Biosystems, USA). Thermocycling conditions included an initial incubation at 50°C for 2 min and at 95°C for 10 min, followed by 45 cycles of 95°C for 15 s and 60°C for 1 min.

Gene expression analyses were performed in the Laboratory of Anatomic Pathology and Molecular Biology (Diagnose Group). The evaluation of the purity and quality of the extracted RNA can be gauged from the good performance in the cDNA quantification and amplification of the chosen reference genes. The cDNA quantification demonstrates the amount of cDNA generated from the extracted RNA following the protocols of the RecoverAll Total Nucleic Acid Isolation Optimized for FFPE Samples kit (Invitrogen-ThermoFisher™). The specifications of the kit inform that the RNA extracted by the methodology is viable for performing RT-PCR and recommend the analysis of small amplicons. Taking this information into account, the quality of the material recovered from the paraffin blocks can be inferred from the amplification of the chosen reference genes *GAPDH* (Hs03929097_g1), with an amplicon of 58 bp, and *ACTB* (Hs99999903_m1), with a larger amplicon, 171 bp.

We assessed DNA repair gene expression using pre-designed Taqman^®^ probes (Life Technologies, USA). The expression of each gene was measured in duplicate and then normalized relative to 2 reference genes: *ACTB*(the gene encoding β-actin) and*GAPDH.* We used normalization based on reference genes to correct differences arising from variation in RNA quality and total RNA quantity in each assay. We used the mean of 2 reference genes to minimize the risk of normalization bias that can result from variations in the expression of any single reference gene. A reference threshold cycle (Ct) for each tested specimen was defined as the average Ct value of the reference genes. The relative mRNA level of a DNA repair gene within a tissue specimen was defined as 2^ΔCt^ ×1000, where ΔCt = Ct (DNA repair gene) − Ct (mean of 2 reference genes).

### Identification of the most stable reference genes

The relative qRT-PCR method requires the use of a normalizing gene as an internal control to correct the differences between the compared samples (19). To this end, we selected 3 well-known reference genes from the literature: *ACTB*, *GAPDH*, and *GUSB*. These genes are constitutively expressed across a wide range of tissues and biological conditions and used as reference genes in the Oncotype DX^®^ assay (9). According to the NormFinder (20), geNorm (21), and BestKeeper (22) statistical algorithms and the ΔCt method (23), *ACTB* + *GAPDH* was the best combination when the 3 candidate reference genes were compared.

Briefly, the NormFinder algorithm first merges group division, absolute gene copy number, and the random expression variation (stability value) caused by biological and experimental factors and then ranks the reference genes in order: the lower the stability value, the more reliable the reference gene (20). The geNorm algorithm calculates a stability value, called M, as the average pairwise variation of each reference gene in relation to all other reference genes, thus allowing the elimination of the least stable gene (21). The BestKeeper algorithm computes the average Ct value, standard deviation (SD), and coefficient of variation for each gene, and genes with an SD >1 are considered unsuitable for use as a stable reference gene (22). The comparative ΔCt method calculates the most stable reference gene from the mean SDs by pairwise comparison of 2 reference genes. An SD <1 indicates stable gene expression (23).

### Definitions

Pathologic complete response (pCR) was defined as disappearance of all invasive cancer in the breast after completion of NACT. Overall survival (OS) was calculated from the date of surgery to the date of death from any cause, or the last date of follow-up. Disease-free survival (DFS) was defined as time from surgery to recurrence or death, whichever occurred first, and was censored at the date of last follow-up for those alive without recurrence. Locoregional recurrence (LRR) was defined as local treatment failure (including relapse on the chest wall, local skin, and operative scar) or regional treatment failure (including recurrence of internal mammary, supraclavicular, and ipsilateral axillary nodes).

### Statistical analysis

We used SPSS 20.0 (USA) for statistical analyses, and a P-value ≤0.05 was considered statistically significant. Survival curves were calculated by the Kaplan-Meier method, and the differences were assessed by the log-rank test. Gene expressions were analyzed by comparing median values using the Mann-Whitney test. Clinicopathological features were analyzed by the Mann-Whitney test or Fisher's exact test.

## Results

Initially, we selected 147 paraffin blocks containing 108 core biopsies and 39 residual tumors after NACT. Five paraffin blocks containing 6 (5.5%) core biopsies and 4 (10.2%) residual tumor were excluded, as they contained a low density of neoplastic cells in relation to the tumor stroma, making it impossible to select a tumor area with safety. In our study, 7 samples did not reach the quality control level and were excluded (4 core biopsy samples and 3 residual tumor samples). In these samples, the Ct values of the reference genes were very different from the mean Ct values of most samples.

We reviewed the medical records of 98 patients with BC treated with NACT. Median patient age was 46.4 (range 24-77) years. Supplementary Table S1 shows the pCR rate according to clinicopathological features.

**Figure 1:**
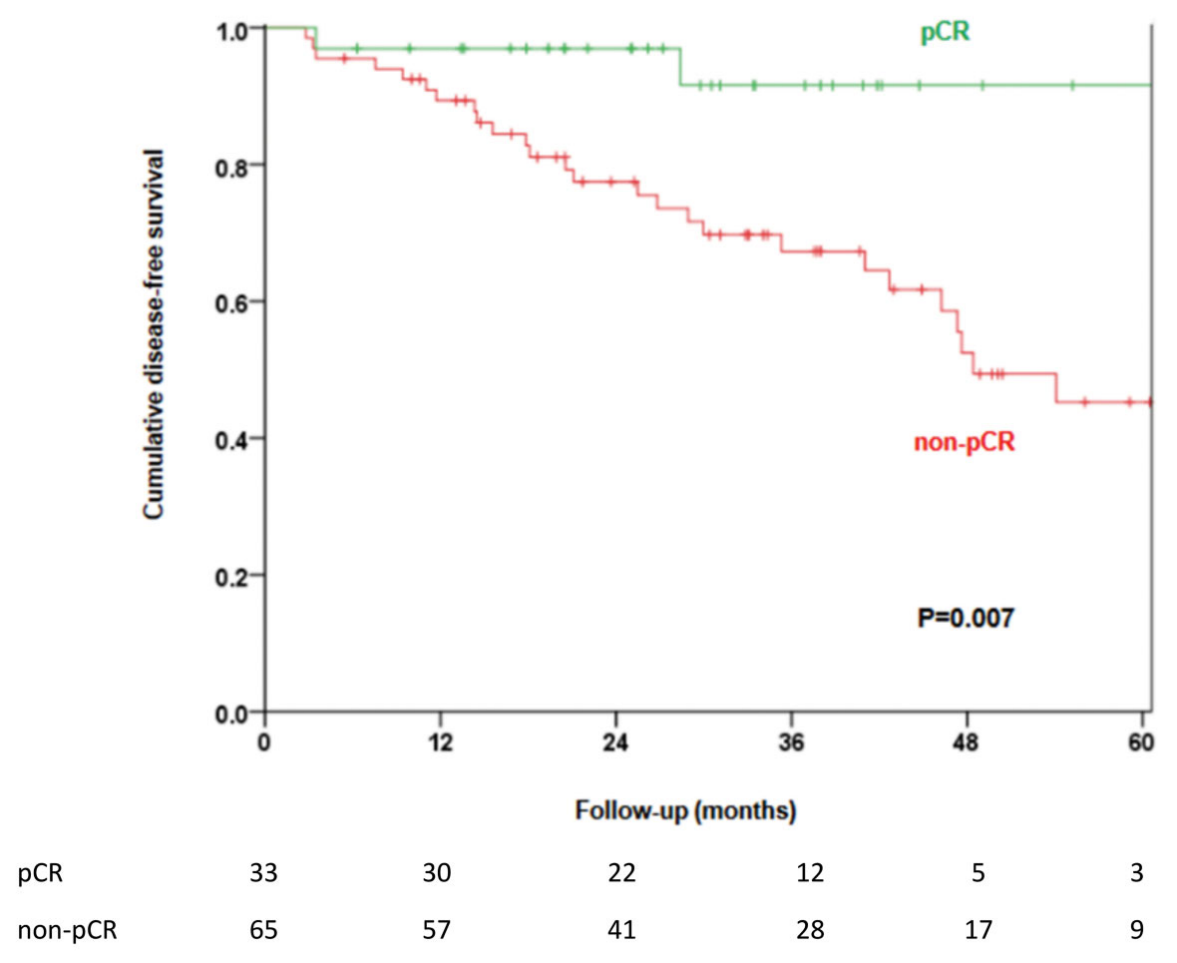
Estimated 5-year disease-free survival by pathologic complete response (pCR) (Kaplan-Meier method). The number of patients at risk at each follow-up time is shown below the graph.

**Figure 2:**
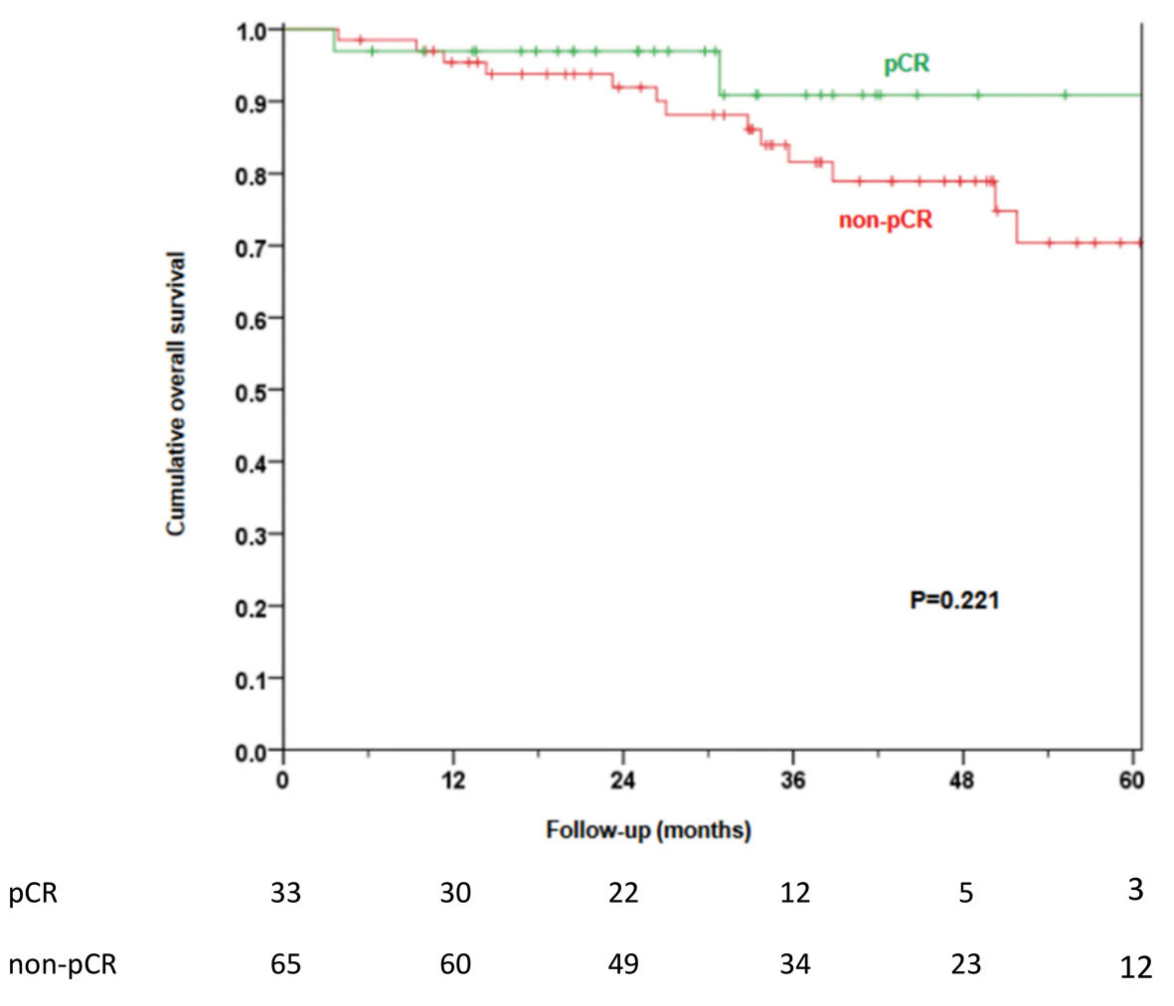
Estimated 5-year overall survival by pathologic complete response (pCR) (Kaplan-Meier method). The number of patients at risk at each follow-up time is shown below the graph.

Overall, 33 patients (33.7%) achieved pCR. The pCR rate was 33.3% (n=2) for luminal A, 18.7% (n=6) for luminal B/HER2-negative, 36.4% (n=8) for luminal B/HER2-positive, 36.4% (n=4) for non-luminal/HER2-positive, and 48.1% (n=13) for triple negative. The tumor progressed during NACT in 10 patients (10.2%). In univariate analysis, histologic grade (P=0.006), PgR expression (P=0.021), and NACT regimen (P=0.03) were significantly associated with pCR. After a median follow-up of 38.5 months, the 5-year cumulative incidence of LRR was 11.8%. The estimated 5-year DFS was 90.9% in the pCR group and 45.3% in the non-pCR group (P=0.007) (Figure 1). Multivariate analysis by Cox regression showed that patients who presented pCR had better DFS regardless of clinical characteristics related to the molecular subtype, clinical stage, and tumor grade (HR=11,0; 95%CI: 2.51 to 48.22; P=0.001). The estimated 5-year OS was 90.9% and 70.4% in the pCR and non-pCR groups, respectively (P=0.221) (Figure 2). Multivariate analysis by Cox regression showed that patients who presented pCR had better OS regardless of clinical characteristics related to the molecular subtype, clinical stage, and tumor grade [hazard ratio (HR)=4.85; 95%CI: 1.04 to 22.60; P=0.044].

### DNA repair gene expression in pre-NACT biopsies

**Figure 3:**
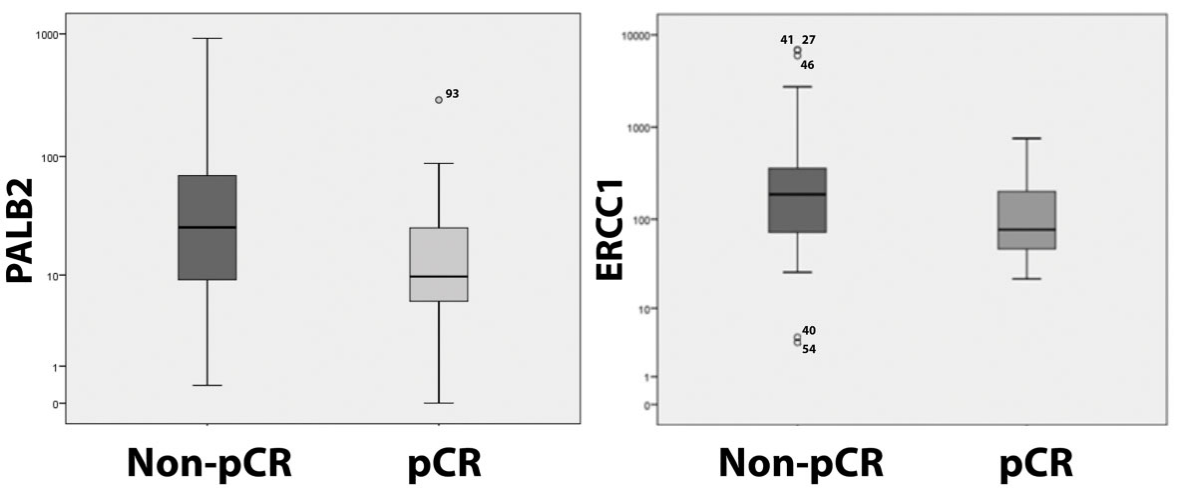
DNA repair gene expression of *PALB2* and *ERCC1* genes in pre-neoadjuvant chemotherapy biopsies by pathologic complete response (pCR). Data are reported as medians (interquartile range). P<0.05 between groups for the two genes (Mann-Whitney test).

Table 1 summarizes the expression of DNA repair genes in pre-NACT core biopsy fragments according to neoadjuvant response. The DNA expression of 2 genes assessed in pre-NACT biopsies (*PALB2* and *ERCC1*) was lower in pCR than in non-pCR patients (P=0.005 and P=0.009, respectively) (Table 1 and Figure 3). After adjustment for tumor grade and molecular subtype, a linear regression model with rank transformation showed that the DNA expression of 2 genes (*PALB2* and *ERCC1*) assessed in pre-NACT biopsies was lower in the pCR group than in the non-pCR group (P=0.014 and P=0.040, respectively).

**Table 1:** DNA repair gene expression in pre-NACT biopsies by pathologic complete response (pCR).

Gene	DNA repair gene expression (percentile)	pCR (n=31) value (×1000)	Non-pCR (n=63) value (×1000)	P*
*BRCA1*	P50	4.81	8.78	0.076
*PALB2*	P50	9.71	25.80	0.005
*RAD51C*	P50	7.46	4.33	0.183
*BRCA2*	P50	2.79	2.84	0.987
*ATM*	P50	21.83	22.14	0.501
*FANCA*	P50	2.79	1.82	0.896
*MSH2*	P50	27.48	38.76	0.274
*XPA*	P50	12.25	16.13	0.075
*ERCC1*	P50	76.94	186.88	0.009
*PARP1*	P50	9.09	7.09	0.384
*SNM1*	P50	16.13	16.75	0.837

NACT: neoadjuvant chemotherapy. *Mann-Whitney test.

### DNA repair gene expression: pre-NACT biopsies vs post-NACT residual tumor

**Figure 4:**
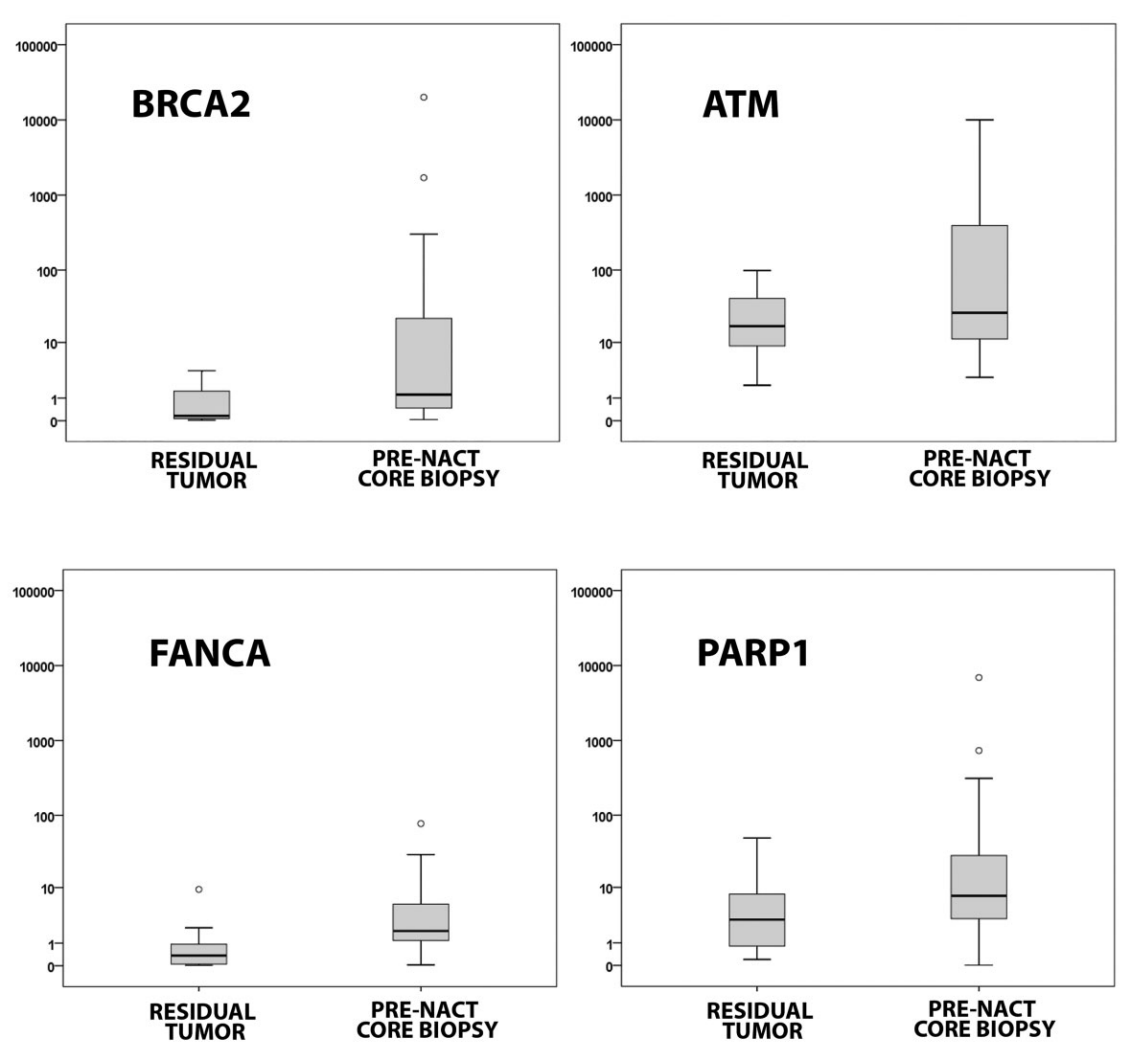
DNA expression of repair genes in post-NACT residual tumors and pre-NACT biopsies. NACT: neoadjuvant chemotherapy. Data are reported as medians (interquartile range). P<0.05 between groups for the four genes (Mann-Whitney test).

The genes *BRCA2* (P=0.009), *ATM* (P=0.004), *FANCA* (P=0.001), and *PARP1* (P=0.011) showed a lower expression in post-NACT residual tumor samples (n=32) than in pre-NACT biopsy samples (n=98) (Table 2 and Figure 4).

**Table 2:** DNA repair gene expression: pre-NACT biopsies *vs* post-NACT residual tumor.

Gene	DNA repair gene expression (percentile)	Core biopsy (n=98) value (×1000)	Residual tumor (n=32) value (×1000)	P*
*BRCA1*	P50	7.66	6.69	0.131
*PALB2*	P50	17.07	24.09	0.166
*RAD51C*	P50	5.42	0.62	0.248
*BRCA2*	P50	2.79	0.16	0.009
*ATM*	P50	21.83	17.11	0.004
*FANCA*	P50	1.85	0.46	0.001
*MSH2*	P50	33.08	42.98	0.218
*XPA*	P50	14.36	24.31	0.395
*ERCC1*	P50	131.93	324.85	0.158
*PARP1*	P50	7.12	2.55	0.011
*SNM1*	P50	16.49	11.51	0.159

NACT: neoadjuvant chemotherapy. *Mann-Whitney test.

## Discussion

DNA repair mechanism consists of several key pathways working together to eliminate DNA lesions and maintain genome stability and integrity (4). Recently developed multiparameter gene-expression assays, mostly based on the expression of genes involved in different DNA repair pathways, have facilitated the selection of patients who are most likely to benefit from systemic chemotherapy (7). In the present study, the DNA expression of 2 genes (*PALB2* and *ERCC1*) assessed in pre-NACT biopsies was lower in the pCR group than in the non-pCR group (P=0.005 and P=0.009, respectively), suggesting that these genes may be predictive markers for NACT response.

The ERCC1 protein plays an essential role in the nucleotide excision repair pathway. The ERCC1-XPF heterodimer cleaves and facilitates the removal of bulky lesions, such as those induced by platinum-based chemotherapy (24,25). *ERCC1* has been well documented as a marker of resistance to chemotherapy in solid tumors other than BC, such as lung, colorectal, head, neck, gastric, bladder, and ovarian cancers (26,27). *ERCC1* expression is an excellent predictor of response to chemotherapy regimens based on anthracyclines and taxanes in patients with early- and advanced-stage BC (7). Low *ERCC1* expression has been associated with higher pCR rates in the NACT setting and with worse OS in patients with hormone receptor-positive BC (7). A prospective study of patients with metastatic triple-negative BC showed that high *ERCC1* expression was associated with worse clinical outcomes for OS and DFS, as well as with a worse response to platinum-based chemotherapy regimens (27).

The *PALB2* gene is vital for homologous recombination repair in response to double-stranded DNA breaks. *PALB2* has been confirmed as a high-risk BC susceptibility gene in recent large-scale analyses of multigene panel testing, with the odds ratio of *PALB2* mutations for BC being comparable to that of *BRCA2* mutations (28,29). Patients with BC harboring the *PALB2* mutation appear to be more likely to present the triple-negative phenotype, advanced disease stage, and higher Ki-67 levels than patients with other familial or sporadic BC (30). Few studies have correlated *PALB2* and chemoresistance. Several deubiquitinases have been implicated as key players in DNA damage repair through homologous recombination. In lung adenocarcinoma, USP22, a deubiquitinase highly overexpressed in multiple cancer types, has been shown to modulate *PALB2* levels through its C-terminal WD40 domain to promote chemoresistance (31). In metastatic BC, germline *PALB2* mutation has been associated with improved disease response to platinum-based chemotherapy (32).

Despite the scarce literature on *ERCC1* and *PALB2* in NACT for BC, our results are consistent with the available data. This highlights the importance of further exploring these DNA repair pathways in the neoadjuvant BC setting. Recently, the I-SPY 2 trial, an adaptive clinical trial platform, was designed to improve outcomes in high-risk breast cancer patients by testing new drugs in the neoadjuvant setting. This study used clinical biomarkers to classify breast cancer into 10 subtypes, allowing individualized patient assignment to therapy arms and maximizing treatment effects (33). The development of specific predictive biomarkers represents the future of breast cancer management and leads to more personalized treatments (33).

In the present study, when comparing the expression of the 11 selected genes between pre-NACT biopsy samples (n=98) and post-NACT residual tumor samples (n=32), we found a lower expression of the genes *BRCA2* (P=0.009), *ATM* (P=0.004), *FANCA* (P=0.001), and *PARP1* (P=0.011) in residual tumor samples. The inactivation of these genes may be directly related to chemoresistance mechanisms, in which cells activate the repair mechanism for damage elimination or proceed toward apoptosis in response to DNA damage by NACT (34). Further studies focusing on the cellular DNA repair mechanism pathways are needed to expand our understanding of BC etiology and to develop therapies specifically targeting the defective pathway in patients with BC (4). This strategy is well established in the treatment of ovarian cancer with *PARP1* inhibitors. *PARP1* is a promising treatment target in BRCA-deficient carcinomas. Homologous recombination-deficient BRCA-mutant carcinomas, which rely on *PARP1*-base excision repair for survival, are highly sensitive to *PARP1* inhibitors through the mechanism of synthetic lethality (35,36).

The strengths of our study include the comprehensive nature of the registry database containing patient characteristics, clinicopathological features, surgery description, adjuvant therapies, and complete ascertainment of patient status at regular follow-up intervals. Another important point is that all gene expression analyses were conducted in a reference center by experts in the field. However, this study also has limitations, including its retrospective nature, small sample size, and heterogeneous population. Furthermore, the high pCR rate for the luminal A subtype may be due to the small number of patients.

### Conclusion

In the era of genomics, treatment should be tailored to the individual patient. The present study showed that *PALB2* and *ERCC1* expressions, assessed in pre-NACT biopsies, were lower in pCR patients. In addition, *BRCA2*, *ATM*, *FANCA*, and *PARP1* expressions were lower in post-NACT residual tumor samples than in pre-NACT biopsies. The use of recently developed multiparameter gene-expression assays, based on the expression of genes involved in different DNA repair pathways, should be further explored in future studies, as they may facilitate the selection of patients most likely to benefit from NACT.

## Supplementary Material

Click to view [pdf].
